# Pattern of Traditional Medicine Utilization among HIV/AIDS Patients on Antiretroviral Therapy at a University Hospital in Northwestern Ethiopia: A Cross-Sectional Study

**DOI:** 10.1155/2017/1724581

**Published:** 2017-03-22

**Authors:** Abyot Endale Gurmu, Fitsum Sebsibe Teni, Wondmagegn Tamiru Tadesse

**Affiliations:** ^1^Department of Pharmacognosy, School of Pharmacy, College of Medicine and Health Sciences, University of Gondar, P.O. Box 196, Gondar, Ethiopia; ^2^Department of Pharmaceutics and Social Pharmacy, School of Pharmacy, College of Health Sciences, Addis Ababa University, Addis Ababa, Ethiopia; ^3^Department of Pharmacology and Clinical Pharmacy, School of Pharmacy, College of Health Sciences, Addis Ababa University, Addis Ababa, Ethiopia

## Abstract

The objective of this study was to assess traditional, complementary, and alternative medicine (TCAM) utilization pattern among HIV/AIDS patients on antiretroviral therapy at University of Gondar Comprehensive Specialized Hospital.* Materials and Methods*. Data on sociodemographic profile and clinical and TCAM utilization were collected using a structured, pretested questionnaire from April 01 to May 28, 2014, through interviews with patients. Data on CD4 count, HIV stage, and ART regimen were collected from patient records. Analysis was conducted descriptively using SPSS version 20.* Results*. Of the 300 participants, 43.7% reported using TCAM, with the largest proportion of them from religious institutions (churches/mosques) (41.22%), followed by home prepared (32.82%) and traditional healers (16.03%). The leading forms of TCAM used were spiritual and herbal therapies constituting 56.49% and 36.64% of the patients, respectively. The most frequently used herbal products included* Nigella sativa *(22.92%) and* Moringa oleifera *(20.83%). Most of the patients (73.30%) using TCAM reported improvement in their conditions.* Conclusions*. TCAM utilization among HIV/AIDS patients on ART was common and different sources and types were used alongside ART, with improvement reported by most. Further research is needed to identify CAM therapies which may be used as adjunct treatments among these patients.

## 1. Introduction

Complementary and alternative medicine (CAM) refers to the use of medical products and practices that are not part of the standard medical care. It can also be described as a health care system that uses accumulated knowledge, skills, and practices obtained from nonformal medical training or practice. Currently, its use is increasing across the world particularly in countries rich with such practices along with the conventional medicine in their national health care system [1].

Patients with HIV/AIDS commonly look for some kind of alternative or complementary therapy parallel to their conventional therapy. Different CAM modalities are taken by HIV/AIDS patients in addition to the principal ART regimen. Herbal or nutritional supplements, mind-body practices, such as yoga or meditation, acupuncture, and spiritual/religious healing comprise the common forms of practices by most patients. In parts of the world where traditional medicine (TM) practice is longstanding and strong, like China, Africa, and India, considerable number of patients visit traditional healers (TH) even before visiting clinics or hospitals providing ART services [2, 3]. Among major reasons that drive patients to seek complementary therapies are unsatisfactory effects, high cost, nonavailability, or adverse effects of conventional medicines [4].

Globally, a number of studies have been conducted to date to assess the level of use, reasons, and effects of traditional, complementary, and alternative medicine (TCAM) on ART. Different studies reported TCAM use along with ART medications by more than half of HIV/AIDS patients, for instance, in Chicago and Ohio, USA, (67%); Thailand (54.7%); KwaZulu-Natal and Pretoria, South Africa (51% and 53%, resp.); Niger Delta, Nigeria (57.9%); Zimbabwe; and Ghana (53.2%). Also, another literature review showed that CAM use among HIV patients ranges from 36 to 68% in Africa [5–13].

In Ethiopia, the practice of TCAM has a very long history according to literatures. It has been employed for the management of various health problems in the country. Evidences show that about 80% of the Ethiopian population is reported to use TCAM in some way. The Ethiopian TM practice encloses different remedies and practices including herbal use, spiritual healing, bone-setting, and minor surgical procedures [14, 15].

According to a study conducted by Kloos et al., 2013; TM practices are commonly employed in the management of HIV/AIDS and HIV related illnesses in Ethiopia either using plant medicines or faith healing. This is associated with the long history, prevailing illness perceptions, and religious beliefs in the country [16].

Despite the observed widespread use of TCAM in the management of HIV/AIDS in Ethiopia, studies documenting this practice among patients on ART are almost nonexistent based on the literature review done. This study is aimed at helping to narrow this gap. The main objective of this study was to assess the TCAM utilization pattern among HIV patients on ART.

## 2. Materials and Methods

### 2.1. Study Setting and Design

A cross-sectional study was conducted from April 01 to May 28, 2014, at the ART clinic of University of Gondar Comprehensive Specialized Hospital. The hospital is located 727 km northwest of Addis Ababa, Ethiopia. It is one of the largest teaching institutions among federally established teaching hospitals and serves about five million populations in and around Gondar town [17].

### 2.2. Sample Size Determination and Sampling Procedure

Convenience sampling technique was used to select patients. All HIV/AIDS patients, who fulfilled the inclusion criteria, that is, 18 years or older, visiting the outpatient department of the ART clinic during the study period and who volunteered to give consent, were included in the study. A total of 300 patients were recruited and participated in the interview of the study.

### 2.3. Data Collection and Management

To extract data, a structured interview questionnaire was administered to the study participants. The questioner contained sections that assessed sociodemographic, clinical characteristics and traditional medicine type and utilization patters. The questionnaire was first prepared in English and translated into Amharic, the local language, and backtranslated to English to ensure that it retained its intended meaning. The questionnaire was pretested to identify potential problem, unanticipated interpretations, and cultural objections to any of the questions on 10 respondents having similar characteristics at the same facility to the study subjects on nonparticipants. The questionnaire was adapted from other similar studies on the use of traditional medicines in HIV/AIDS patient, with a little modification to suit the Ethiopian context [7, 9–11, 13].

Besides, data related to CD4 count, HIV staging, and type of ART regiment were collected from individual medical records. The data collection was conducted by the investigators using the structured questionnaire at the specified setting from April 01 to May 28, 2014.

### 2.4. Data Entry, Analysis, and Interpretation

The data collected in the present study were entered to and analyzed using Statistical Packages for Social Sciences (SPSS) version 20. In the analysis, frequencies and percentages were used in the description of the data collected [18].

### 2.5. Ethical Considerations

Ethical approval was obtained from the ethical review committee of School of Pharmacy, College of Medicine and Health Science, University of Gondar, and letter of permission was obtained from the hospital ART clinic based on the request of the school. Besides, study participants were asked for informed oral consent and their information was maintained confidentially.

## 3. Results

### 3.1. Sociodemographic Profiles of Respondents

A total of 300 HIV/AIDS patients were interviewed and included in the analysis. About 173 (57.7%) of the study participants were females and 111 (37.0%) fall in the age range of 36–45 years. On the other hand, more than three-quarters (77.7%) of the study participants were Ethiopian Orthodox Christians by religion. Nearly half (48.3%) of the participants were married and about 26.7% were literate (able to read and write). With regard to employment status, about 58.3% were employed and about one-fourth (25.7%) earned less than 100 Ethiopian Birr (ETB) per month during the study period. Majority (79%) of the respondents were from urban areas (Table 1).

### 3.2. Clinical Characteristics of the Participants

Nearly two-thirds (63.7%) of the participants knew their HIV status some 2 years back from the date of data collection period. More than half (55.3%) of the patients were taking combinations of 1C (Zidovudine (AZT) + Lamivudine (3TC) + Nevirapine (NVP)) and just above half of the patients had a CD4 count of more than 350 cells/microliter (Table 2).

Nearly two-thirds (63.7%) of the patients were in clinical stage I of the disease while only 4.7% were in stage IV. Cotrimoxazole was found to be the most frequent medicine being taken by more than half (52.66%) of the patients in addition to the combination ART medications to deal with opportunistic infections and symptoms associated with HIV/AIDS. On the other hand, 40% of the patients took exclusively ART mediations with no additional medications (Table 2).

### 3.3. Utilization of Traditional Medicine

In this study, nearly half (43.7%) of the participants reported that they used TCAM for the management of HIV/AIDS among which more than half (52.7%) reported that they began TCAM use after the initiation of ART (Table 3).

With regard to the sources of TCAM modalities, the majority of patients who used TM reported that their sources were religious institutions (churches/mosques) (41.22%), followed by homemade preparations (32.82%) and preparations from traditional healers (16.03%). The leading forms of TCAM used by the patients were spiritual therapy (56.49%) and herbal therapy (36.64%) (Table 3). The study showed that most patients use TM because of various motivating reasons such as religious practices (38.93%), need of improving immunity (22.90%), and recommendations from family members (19.08) (Table 3).

The most frequently used herbal products, alongside ART regimens, included* Nigella sativa* (22.92%),* Moringa oleifera* (20.83%), and other herbs/herbal recipes most commonly prescribed by traditional healers (TH) (18.75%) (Table 4).

**Figure 1:**
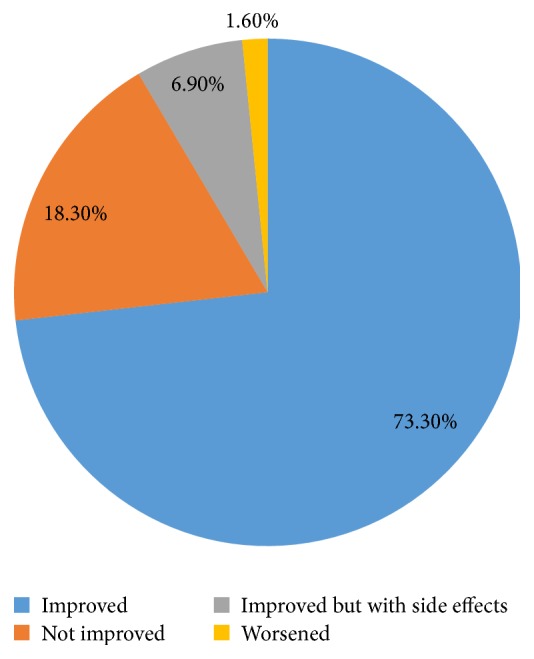
Percentage distribution of reported outcomes of TM use by HIV/AIDS patients in ART clinic, May 2014.

About three-quarters (73.30%) of the patients who used TM reported that they had improved disease condition while nearly one-fifth indicated they did not (Figure 1).

With regard to counseling of TM preparation use along with ART medications by health care providers, about 39% of the participants reported that they received counseling. However, more than half (53.7%) of the patients reported that they thought no interaction exists between TM and ART, whereas about 6.7% of patients who use TCAM reported that they had experienced problems when taking TM and ART simultaneously.

## 4. Discussion

The study revealed that nearly half of the patients in the study reported using TM besides ART regimens. A similar study in Thailand which involved 160 participants showed that 95% of patients with HIV used complementary and alternative medicines (CAM) and 78% visited a CAM provider [8].

A cross-sectional study in Ghana, on the other hand, reported about 53.2% of HIV/AIDS patients in the study used TM which is more or less comparable to this study. In a similar study in British Columbia, Canada, nearly half (47%) of participants in the study had ever used CAM which is almost similar proportion to this study. In another study done in KwaZulu-Natal, South Africa, the proportion of HIV AIDS patients who used traditional complementary and alternative medicine (TCAM) was 51.3% in the previous six months before the study [7, 13, 19].

On the other hand, the type of commonly used TM in the present study was spiritual therapy followed by herbal therapy. A similar pattern was shown by a study done in Ghana where herbal therapy was the most frequently used one by 70% of the TM users. However, in the Thailand study spiritual therapy was reported as the most commonly used CAM modality with 84% of the CAM users. In the Canada study the types of CAM commonly used differ from this study where vitamins/minerals (81%), meditation/yoga (36%), massage (31%), marijuana (30%), dietary supplements (24%), and herbal medicines (19%) were among the listed forms of CAM [8, 13, 19].

According to the findings of this study, taking TM as part of religious practice (38.93%), the belief and desire by patients to improve their immune system (22.90%) and recommendation from family members were the motivating factors for TM use in HIV/AIDS patients. In the study done in Ghana, it was reported that TM was practiced among HIV/AIDS patients mainly for appetite (90.9%), pain relief (87.9%), stress relief (63.6%), and general wellbeing (75.8%). Based on literatures in the area it is established that CAM is commonly used as an adjunct to ART and HIV-infected people and AIDS patients often seek complementary therapies including herbal medicines due to reasons such as unsatisfactory effects, high cost, nonavailability, or adverse effects of conventional medicines. Particularly in Africa, TM and natural medicines have been employed as primary HIV treatment and for HIV related symptoms including dermatological disorders, nausea, depression, insomnia, and weakness [5, 7, 13, 20].

In the current study nearly three-quarters of the patients taking TM reported to have had improved disease condition after the initiation of the therapy. A study done in China on seventy-six male HIV patients on use of traditional Chinese medicine showed that all except one had achieved undetectable viral load [21].

Among the participants of the study nearly half reported that they thought TM and ART have interaction. In addition, a considerable proportion of patients (6.7%) using TM in this study reported to experience problems when using ART and TM concurrently. These negative effects can result from lack of communication between patient and health care provider as 61% of our respondents did not get advice about TM. The study in South Africa on TM showed there is a potential for ARV nonadherence and serious drug interactions among patients with HIV/AIDS. Similarly, the study in Ghana cited above reported that concomitant TM use with ART has a propensity for drug interactions. Yet another study in Zimbabwe reported some traditional herbal medicines may increase incidence of certain types of adverse events when used with ART [7, 12, 13].

A study done on the TM of Ethiopia reported that consumption of herbs and spices as part of a normal diet is not likely to cause adverse herb-drug interactions because they are consumed in some relatively small quantities. However, when these are used for medicinal purposes, the study reported that it might increase the likelihood of adverse interactions with conventional medicines [14].

As shown in Table 5, previous studies also showed that* Nigella sativa, Allium sativum*,* Zingiber officinale*, and* Moringa oleifera* are used elsewhere as immunomodulator, antioxidant, anti-inflammatory, analgesic, and antimicrobial agents. This bioactivity might benefit HIV/AIDS patients to boost the immunity, to scavenge free radicals, and to fight against opportunistic infections.

## 5. Limitation

Being a cross-sectional design conducted at a single university hospital, our study findings cannot be generalized to all areas of the country. Like most interview based researches, the study findings might be affected by the respondents' willingness to provide correct information and the way they understand interview questions. Moreover, the small sample size utilized in this study and the convenience sampling might also affect representativeness to all patients on ART in the hospital.

## 6. Conclusion

TM use among HIV/AIDS patients who were on ART was a common practice, according to the results of this study. Spiritual therapy and herbal therapy were the most frequently used TM modalities among the study population. Majority of TM users reported improvement after using it though some proportion of patients complained about problems associated with concurrent use of TM with ART. Further investigation should be done to identify CAM therapies that may be used as adjunct treatments in patients with HIV/AIDS.

## Figures and Tables

**Table 1:** Sociodemographic characteristics of the respondents, May 2014.

Variable	Number (%)
*Sex*	
Male	127 (42.3)
Female	173 (57.7)
*Age (years)*	
18–25	36 (12.0)
26–35	109 (36.3)
36–45	111 (37.0)
46+	44 (14.7)
*Religion*	
Orthodox Christian	233 (77.7)
Muslim	48 (16.0)
Protestant	16 (5.3)
Catholic	3 (1.0)
*Marital status*	
Unmarried	55 (18.3)
Married	145 (48.3)
Divorced	62 (20.7)
Widowed	38 (12.7)
*Educational status*	
Not able to read and write	61 (20.3)
Able to read and write	80 (26.7)
Primary school	48 (16)
Secondary school	57 (19.0)
College and above	54 (18.0)
*Residential area*	
Urban	63 (21.0)
Rural	237 (79.0)
*Monthly income (ETB)*	
<100	77 (25.7)
100–300	35 (11.7)
301–500	51 (17.0)
501–800	36 (12.0)
801–1000	33 (11.0)
>1000	68 (22.7)
*Occupational status*	
Housewife	40 (13.3)
Unemployed	51 (17.0)
Employed	175 (58.3)
Pensioner	16 (5.3)
Student	16 (5.3)
Commercial sex workers	2 (0.7)

**Table 2:** Frequency distribution of clinical characteristics of the respondents, May 2014.

Variable	Frequency (%)
*Time since patient was diagnosed to have HIV*	
<1 year	40 (13.3)
1-2 years	69 (23.0)
>2 years	191 (63.7)
*CD4 cell count (count/microliter)*	
<100	32 (10.7)
100–350	110 (36.7)
>350	158 (52.7)
*Clinical stage *	
Stage I	189 (63.0%)
Stage II	53 (17.7%)
Stage III	44 (14.7%)
Stage IV	14 (4.7%)
*ART regimen*	
AZT + 3TC + NVP	166 (55.3%)
TDF + 3TC + NVP	53 (17.7%)
TDF + 3TC + EFV	40 (13.3%)
AZT + 3TC + EFV	32 (10.7%)
Other	9 (3.0%)
*Medicines other than ART*	
Cotrimoxazole	158 (52.66%)
Fluconazole	9 (3.0%)
Amoxicillin	5 (1.5%)
Oral contraceptives	2 (0.7%)
Anti TB	2 (0.7%)
Antimalaria	1 (0.3%)
Ibuprofen	1 (0.3%)
Sodium valproate and phenobarbitone	2 (0.7%)
Ephedrine	1 (0.3%)
Only ART	119 (40.0%)

**Table 3:** The utilization pattern among HIV patients on ART using TCAM, May 2014.

Variable	Frequency (%)
*Time of TCAM start*	
Before ART initiation	62 (47.30)
After ART initiation	69 (52.70)
*Motivating factors for using TCAM*	
Family	25 (19.08)
Health care provider	5 (3.82)
Religion	51 (38.93)
To improve immunity	30 (22.90)
To deal with side effects of ART	2 (1.53)
Additional benefit	16 (12.21)
Other	2 (1.53)
*Type of TCAM*	
Herbal therapy	48 (36.64)
Spiritual therapy	74 (56.49)
Body-mind therapy	3 (2.29)
Others	6 (4.58)

**Table 4:** Types of herbal medicines taken by the patients using TCAM in the hospital, May 2014.

Type of herbal medicine used	Frequency (%)
Mixture of spices	4 (8.33)
Black cumin *(Nigella sativa)*	11 (22.92)
Ginger *(Zingiber officinale)*	5 (10.42)
Garlic *(Allium sativum)*	6 (12.5)
Moringa *(Moringa oleifera)*	10 (20.83)
Unspecified herbal products	12 (25)

**Table 5:** Previous studies on the use of reported medicinal plants.

Plant name	Plant part/type of preparation	Medicinal use	References
Black cumin *(Nigella sativa)*	Volatile oil of the seed	Immunomodulatory, anti-inflammatory, analgesic, antipyretic, antimicrobial, antineoplastic, and antioxidant activity	[22–24]
Garlic *(Allium sativum)*	Isolated compounds (Ajoenes), crude extract of the bulb	Activities against immunodeficiency virus, antimicrobial, antioxidant, anticarcinogenic, and antioxidants activities	[25–28]
Ginger* (Zingiber officinale)*	Methanolic extracts of the leaves and rhizomes	Immunomodulatory, antitumorigenic, anti-inflammatory, antiapoptotic, antihyperglycemic, antilipidemic antiemetic, antioxidants, and antimicrobials activities	[29, 30]
Moringa* (Moringa oleifera)*	Volatile oil from leaves; extracts from leaves and roots	Anti-HSV (herpes simplex virus type 1), antioxidant, antimicrobial, antihypertensive, diuretic, cholesterol lowering, antitumor, and anticancer activities	[31–33]
